# Toward precision prescribing for methadone: Determinants of methadone deposition

**DOI:** 10.1371/journal.pone.0231467

**Published:** 2020-04-17

**Authors:** Andrew H. Talal, Yuxin Ding, Charles S. Venuto, Lindsay M. Chakan, Anthony McLeod, Arpan Dharia, Gene D. Morse, Lawrence S. Brown, Marianthi Markatou, Evan D. Kharasch

**Affiliations:** 1 Division of Gastroenterology, Hepatology and Nutrition, Jacobs School of Medicine and Biomedical Sciences, University at Buffalo, Buffalo, NY, United States of America; 2 Department of Biostatistics, School of Public Health and Health Professions, University at Buffalo, Buffalo, NY, United States of America; 3 Department of Neurology, University of Rochester, Rochester, NY, United States of America; 4 START Treatment & Recovery Centers, Brooklyn, NY, United States of America; 5 NYS Center of Excellence in Bioinformatics and Life Sciences, University at Buffalo, Buffalo, NY, United States of America; 6 Department of Anesthesiology, Duke University School of Medicine, Durham, NC, United States of America; Harvard Medical School, UNITED STATES

## Abstract

**Background:**

Despite the World Health Organization listing methadone as an essential medication, effective dose selection is challenging, especially in racial and ethnic minority populations. Subtherapeutic doses can result in withdrawal symptoms while supratherapeutic doses can result in overdose and death. Although CYP3A4 was conventionally considered the principal methadone metabolizing enzyme, more recent data have identified CYP2B6 as the principal enzyme. CYP2B6 has ethnically-associated polymorphisms that affect the metabolic rate. Our objective was to investigate the effects of genetic and nongenetic factors on methadone metabolism.

**Methods:**

We measured trough plasma methadone levels in 100 participants with opioid use disorder. We assessed methadone metabolism by calculating the metabolite ratio (major metabolite: 2-ethylidene-1,5-dimethyl-3,3-diphenylpyrrolidine [EDDP] divided by methadone concentration). We assessed hepatic fibrosis and steatosis by transient elastography and C*YP2B6* alleles, principally responsible for methadone metabolism. Mixed effects models modeled the data in 97 participants.

**Results:**

Participants were largely male (58%), minority (61% African American) and non-Hispanic (68%). Forty percent were HCV mono-infected, 40% were uninfected, and 20% were HCV/HIV co-infected. Female sex had significant effects on (R)- and (S)-methadone metabolism (p = 0.016 and p = 0.044, respectively). *CYP2B6* loss of function (LOF) alleles significantly affected (S)-methadone metabolism (p = 0.012). Body mass index (BMI) significantly affected (R)-methadone metabolism (p = 0.034). Methadone metabolism appeared to be lower in males, in individuals with LOF alleles, and elevated BMI.

**Conclusions:**

Genetic analysis, especially in minority populations, is essential to delivering individualized treatments. Although the principal methadone metabolizing enzyme remains controversial, our results suggest that sex, *CYP2B6* genotype, and BMI should be incorporated into multivariate models to create methadone dosing algorithms. Methadone dosing algorithms should facilitate medication delivery, improve patient satisfaction, and diminish overdose potential.

## Introduction

Since the 1960s, medication-assisted treatment has been the standard therapy for opioid use disorder (OUD) [1]. Methadone, a synthetic opioid, blocks the euphoric effects of opioids while relieving physiological cravings and alleviating withdrawal symptoms. The narrow therapeutic index and high inter-individual pharmacokinetic (PK) variability of methadone create dosing challenges. Methadone overdoses may lead to toxicity and death; subtherapeutic doses may potentiate withdrawal and necessitate ongoing opioid use in order to minimize breakthrough withdrawal symptoms. Persistent withdrawal symptoms (e.g., nausea) can lead to ongoing illicit opioid use in conjunction with methadone, which can result in death. Thus, therapeutic dosing of methadone requires low dose initiation, careful dose titration, and diligent monitoring for signs of withdrawal or overdose [2–4]. The current methadone-dosing scheme relies principally on dose titration, which is very time consuming and complex from both the patient and provider standpoints. With increasing methadone prescription, an urgent need exists for an enhanced understanding of methadone PK in order to develop refined dosing strategies to ultimately reduce morbidity and mortality [5].

Methadone prescribed for humans is comprised of two enantiomers, R and S; large variation in individual PK between (R)- and (S)-methadone creates additional dosing challenges. (R)-methadone has a 10-fold higher affinity for μ-opioid receptors, mediating much of the drug’s clinical effect, while the affinity of (R)- and (S)-methadone for NMDA receptors is similar [6, 7]. Although the major methadone metabolizing enzyme remains controversial, recent data suggest that cytochrome P4502B6 (CYP2B6) is thought to be the major hepatic enzyme responsible for methadone metabolism [8–12]. Other hepatic enzymes including CYP3A4 and CYP2D6, however, have been purported to also play a role [13, 14]. Limited information, however, exists on the effects of *CYP2B6* genetic polymorphisms on methadone metabolism, especially in minority populations. Various *CYP2B6* alleles have been associated with changes in methadone metabolism [15]. One of the earliest identified allelic variants, CYP2B6*6, has been associated with reduced methadone metabolism [16].

Additional potentially important nongenetic factors in methadone metabolism are those that might affect liver function, such as fibrosis stage, steatosis severity, infection with HIV and/or hepatitis C virus (HCV), concomitant medications, and body mass index (BMI). Both HIV and HCV incidence has increased recently as a result of the opioid epidemic [17, 18]. HCV results in liver fibrosis and liver dysfunction that may lead to cirrhosis and hepatocellular carcinoma [19, 20]. Furthermore, both HIV and HCV have been associated with hepatic steatosis, as has metabolic dysfunction [21–23]. Crucial to the development of improved methadone dosing strategies is understanding both the genetic and nongenetic factors that affect its metabolism [24].

We sought to elucidate the effects of *CYP2B6* allelic variability and chronic liver disease due to opioid-related infections on methadone PK in a predominantly minority population of OUD patients on methadone. We assessed the ratio of (R)- and (S)- plasma methadone metabolite (2-ethylidene-1,5-dimethyl-3,3-diphenylpyrrolidine, EDDP) to parent drug concentrations as a measure of methadone metabolism. We enrolled 100 eligible OUD participants on methadone from a population consisting primarily of racial and ethnic minorities. We hypothesized that 1) *CYP2B6* loss of function (LOF) alleles would result in decreased methadone metabolism and 2) *CYP2B6* LOF alleles are more common in racial and ethnic minorities. We also investigated the relationships between methadone metabolism and hepatic fibrosis stage as well as the degree of steatosis.

## Materials and methods

MeDiCALF (Methadone Disposition Changes Associated with Liver Fibrosis) was a cross-sectional community-based, point-of-care study that enrolled a total of 100 HIV/hepatitis C virus (HCV) co-infected, HCV mono-infected, and participants without HIV or HCV infections (i.e., uninfected individuals) receiving stable, once-daily oral methadone for OUD. A total of 97 participants were included in the final analysis.

### Participants

The study protocol was approved by the institutional review board at START Treatment & Recovery Centers, the University of Rochester, and the University at Buffalo. Inclusion criteria required that study participants were at least 18 years of age and were actively enrolled at START Treatment & Recovery Centers for at least 90 days prior to study entry, after the intensive stabilization phase [25]. Participants must have received stable, once-daily methadone for at least 14 days prior to study entry with at least 80% methadone adherence. Exclusion criteria included active hepatitis B virus infection, ongoing treatment for HCV infection, pregnancy/breastfeeding, mental instability that might interfere with completion of study related activities, or factors that would preclude performance of vibration-controlled transient elastography (VCTE) such as ascites, BMI greater than 40 kg/m^2^, or implantable medical devices.

START Treatment & Recovery Center assesses HIV and HCV serology on an annual basis with HIV or HCV RNA obtained at least initially on all seropositive individuals. Infection status was determined through data contained in the electronic medical record (EMR). If HIV and/or HCV serological testing did not occur within 365 days from screening, the study staff ordered these tests. HIV positivity was defined as testing positive for HIV at any time. For HIV-infected individuals, the date of HIV diagnosis was recorded, along with the most recent HIV RNA level and CD4^+^ cell count. HCV positivity was defined as HCV RNA positivity within 365 days from screening. HCV-infected participants had to be HCV treatment-naive or to have had documented unsuccessful prior treatment with HCV antivirals. Uninfected participants were seronegative for both HIV and HCV.

Informed consent was obtained on 100 individuals and 97 were included in the analysis. Reasons for exclusion from the analysis of the three participants were as follows: one participant with BMI >40 kg/m^2^ was excluded due to inability to perform valid VCTE measurements, one participant did not have genotyping information available, and one participant was excluded from the statistical modeling due to prescription of medications (i.e., clopidogrel) that inhibit CYP2B6 resulting in insufficient numbers of participants in this category to obtain meaningful results.

Medication history included medication doses as well as the start/stop dates for the following: antiretroviral therapy, HCV treatment, methadone, and prescription and non-prescription drugs. Drug interactions were categorized based on the effect on CYP2B6 enzymatic activity as inducers or inhibitors. CYP2B6 inducers included efavirenz [26, 27], carbamazepine [28] and nelfinavir (via CYP2B6 induction and increase in renal clearance [29]). Clopidogrel was classified as a CYP2B6 inhibitor [30].

### Data collection

Participants were identified by participant identification numbers in the case report form. Only research study staff had access to participant’s data. All laboratory specimens, evaluation forms, and other records were identified by coded numbers only in order to maintain participant confidentiality. This included samples for genetic testing that were de-identified, provided that the participant had consented to permit genetic testing.

### Clinical assessments

Potential participants were identified through START Treatment & Recovery Center’s EMR. Potential participants were subsequently approached and requested to sign informed consent. Screening evaluations to determine study eligibility were completed within 30 days of obtaining informed consent and study enrollment. Demographic information included sex, race, ethnicity and age. Height and weight were documented at screening and at study entry from the EMR. Onsite, rapid pregnancy tests were performed in all female study participants of childbearing age. For HCV/HIV co-infected individuals, the most recent HIV RNA level and CD4^+^ cell count were recorded.

A single pre-dose methadone trough pharmacokinetic (PK) venous blood sample was drawn at study entry, within 1 hour prior to the participant’s scheduled dose of methadone. After collection, blood was centrifuged, plasma removed, and stored at -20°C until processing. The times of the prior methadone dose, blood sample collection, and methadone dose administration were recorded on the case report form (CRF).

The Clinical Opioid Withdrawal Scale (COWS) is an 11-item scale used to rate symptoms of opiate withdrawal as well as to assess the severity of physical dependence. The scale includes assessment of resting heart rate, restlessness, sweating, skin changes, pupil size, runny nose, teary eyes, yawning, body aches, gastrointestinal discomfort, tremor, anxiety and irritability [31]. The individual items in the COWS are summarized and interpreted as follows: score <5, no withdrawal; 5 to 12, mild withdrawal; 13 to 24, moderate withdrawal; 25 to 36, moderately severe withdrawal; more than 36, severe withdrawal.

The Opiate Overdose Assessment (OOA) is an 11-item scale used to assess symptoms of opioid overdose (Jerry Friedland, MD, personal communication). The scale consists of 8 patient reported symptoms (sweating, slurred speech, nausea, vomiting, tremor, fatigue, incoordination, and slow breathing) and 3 clinically-reported signs (respiratory depression, pinpoint pupils, and drowsiness) as assessed by a clinician. Patient reported symptoms were graded as: 0, not at all; 1, slight; 2, moderate; 3, quite a bit; 4, extreme. Patient symptoms were summed and a total score out of 32 was provided as well as a report of the individual clinically evaluated signs [32]. Both scales were administered at study entry, prior to the participant’s daily methadone dose.

### Liver fibrosis and steatosis measurement

VCTE provides Liver Stiffness Measurement (LSM) and Controlled Attenuation Parameter (CAP) values as assessed using FibroScan® (FS Compact 530-Echosens, Paris, France). LSM is correlated to liver fibrosis stage and CAP is correlated to liver steatosis grade [33, 34]. Exclusion criteria for VCTE included pregnancy, presence of an implantable medical device, and BMI>40. Consistent with standard practice for VCTE, participants were consented and requested to fast for 3 hours before the procedure. The examination was performed onsite in the methadone program with participants positioned supine with the right leg over the left leg, and the right arm over the head. The ultrasound-like probe was placed on the skin over the liver area, typically in the right mid-axillary line. The probe generated mechanical waves that moved toward and through the liver [35]. After a minimum of 10 valid measurements were acquired, median LSM and CAP values were calculated [36]. Thus, the LSM and CAP measurements are median measurements. Examinations with an interquartile range greater than 30% were classified as unreliable and were excluded from further analysis [37]. The HCV mono-infected cutoff values for fibrosis stage were: LSM ≤7.0 kPa, F0-F1; LSM>7.0 kPa, F2; LSM≥9.5 kPa, F3; LSM≥12.0 kPa, F4. The HCV/HIV co-infected cutoff values for fibrosis stage were: LSM≤7.0 kPa, F0-F1; LSM≤10.0 kPa, F2; LSM≥11.0 kPa, F3; LSM≥14.0 kPa, F4. The uninfected cutoff values for fibrosis stage were: LSM<10 kPa, F0-F2; LSM ≥10 kPa, F3-F4 [33]. The cutoff values for steatosis grade were: CAP>248 dB/m, S1; CAP>268 dB/m, S2; CAP>280 dB/m, S3 [38, 39]. VCTE provided a measure of liver stiffness (lower kPa values indicate a more elastic liver), a surrogate measure for fibrosis. CAP provided an indicator of steatosis (lower dB/m values indicate less steatosis).

### Participant compensation

Participants were compensated $50 to complete an examination, including obtaining informed consent and for responding to the COWS and OOA. We also compensated participants $50 for VCTE and $25 for blood draws. Participants who completed all study related procedures were compensated a total of $125.

### Genotyping

Whole blood samples for *CYP2B6* genotyping were analyzed as previously described [11]. Genomic DNA was extracted from 400 L whole blood collected in EDTA using the DSP DNA Midi Kit in combination with the QiaSymphony automated system (QIAGEN, Valencia, CA, USA). Samples were stored at -20°C until analysis. Each sample was genotyped for the *CYP2B6* 516G>T (rs3745274), 785A>G (rs2279343), 983T>C (rs28399499) and 1459 C>T (rs3211371) single nucleotide polymorphism (SNP). Genotyping was performed using Agena iPLEX (Agena San Diego, CA, USA) and validated on the Coriell SNP 500 DNA set and CEPH trios, at the Genomics Shared Resource at Roswell Park Comprehensive Cancer Center in Buffalo, NY. Primer sequences were designed as previously described [11]. Analysis of the SNPs permitted the detection of CYP2B6*1, CYP2B6*4 (785A>G), CYP2B6*5 (1459C>T), CYP2B6*6 (516G>T, 785A>G), CYP2B6*7 (516G>T, 785A>G, 1459C>T), CYP2B6*9 (516G>T), CYP2B6*16 (785A>G, 983T>C) and CYP2B6*18 (983T>C) alleles. Based on an individual’s *CYP2B6* genotype, a metabolizing phenotype status was determined as follows: normal function: *1/*1, *1/*5, *1/*7; LOF: *1/*6, *1/*18, *6/*16, *6/*6; and gain of function: *1/*4, *4/*6 [40].

### Methadone assay

We measured (R)- and (S)-methadone concentration (ng/mL) and (R)- and (S)-EDDP concentration (ng/ml) as well as calculated methadone metabolism by the (R)- and (S)-EDDP/methadone concentration ratio. Plasma methadone and (R)- and (S)-EDDP metabolite enantiomer concentrations were determined using chiral liquid chromatography-tandem electrospray mass spectrometry as previously described [12]. Interday coefficients of variation for 5, 20, 200 ng/mL (R)- and (S)-methadone were 7, 4 and 17% and 5, 4 and 8%, respectively. Interday coefficients of variation for 1 and 5 ng/mL (R)- and (S)-EDDP were 3 and 6% and 5 and 13%, respectively. No samples required re-assay.

### PK analysis

The study design and setting did not require study participants to receive a specific uniform dose of methadone. Therefore, methadone dosing was variable across study participants. For the purpose of presenting the pharmacokinetic data, the (R)- and (S)-methadone and (R)- and (S)-EDDP plasma concentrations were dose-normalized using the free-base enantiomeric methadone dose (not the total, or HCl salt dose).

### Statistical analysis

A total of 100 participants provided informed consent and 97 were included in the analysis. To evaluate data distributional characteristics and associations between explanatory and outcome variables, exploratory and correlation analyses were performed. Correlation analysis initially evaluated the association between explanatory variables and outcome variables.

#### Outcome/response variables

The (R)- and (S)-EDDP/methadone concentration ratios are the primary outcome variables. We used ln([R]-EDDP/methadone concentration) and ln([S]-EDDP/methadone concentration) in the analysis. The logarithmic transformation with natural basis is denoted as ln(.), and it was used to correct for symmetry.

#### Correlation analysis

Correlation analysis initially evaluated the association between explanatory variables and outcome variables. Spearman’s rank correlation coefficient is a non-parametric rank-based correlation measure between two continuous random variables [41]. Rank-Biserial correlation coefficients are calculated when there is one continuous variable and one dichotomous variable [42, 43]. A strong correlation is defined as a correlation coefficient greater than 0.5 or less than -0.5.

#### Linear regression

Linear regression was applied to obtain the fitted lines and estimates of parameters between methadone dose and methadone concentration as well as methadone metabolism. Robust linear regression is applied to obtain the fitted lines and estimates of parameters in the presence of outliers. R Version 3.5.2 (R Core Team, Vienna, Austria) function “lm” was used for linear regression and “rlm” was used for robust linear regression. In the robust linear regression, Huber weights were created by the iterated re-weighted least squares (IRLS) process [44]. The tuning constant of Huber regression is 1.345. Asymptotically, it provides 95% efficiency, similar to that obtained with linear regression when applied to the normal distribution.

#### Modeling

We fit a multivariate linear mixed effects model using SAS Version 9.4 (SAS Corporation, Cary, NC), in which the fixed effects include the primary variables of interest (sex, BMI, *CYP2B6* genotype, concomitant medications, and the interaction between concomitant medications and *CYP2B6* genotype). The random effects include a random intercept, which indicates the inter-individual variability among participants with different infection status nested in different sites, and a random effect due to sites. The model takes into account 1) the variables of primary interest, 2) the manner in which individual participants were sampled, 3) the correlation between (R)- and (S)-EDDP/methadone concentrations, and 4) the sample size of the population enrolled.

For categorical explanatory variables, the normative category is selected as the reference category. Participants with normal function alleles are the reference category for genotype, participants taking no concomitant CYP2B6 inducer medications are the reference category for concomitant CYP2B6 inducer medication status, and male individuals are the reference group for sex.

The participants were either HCV mono-infected, HCV/HIV co-infected or uninfected. The distribution of participant’s infection status derived from each clinic is shown in Table 1. The significance level for all analyses was set at 0.05.

**Table 1:** Distribution of participants by recruitment site and infection status.

Site ID Number	Infection Status			Total
	HCV/HIV	HCV-mono	Uninfected	
2	3	7	9	19
3	6	19	21	46
4	1	7	3	11
5	1	6	6	13
6	5	0	0	5
8	3	0	0	3

Abbreviations: ID, identification; HCV, hepatitis C virus; HIV, human immunodeficiency virus.

## Results

### Demographic characteristics

A total of 97 participants were included in the final analysis, including 19 HCV/HIV co-infected (20%), 39 HCV mono-infected (40%) and 39 uninfected (40%). The majority were male (58%), African-American (61%), and non-Hispanic (68%) (Table 2). The median age was 56 years (interquartile range [IQR] 14) and the uninfected group was the youngest (median age 53, IQR 11).

**Table 2:** Demographic and medical characteristics of the study participants. The table presents counts and associated percentages or mean, median and associated standard deviation (SD) or interquartile range (IQR) as appropriate.

	Variable	Infection status			
		HCV-mono	HIV/HCV	Uninfected	Total
		n = 39 (40%)	n = 19 (20%)	n = 39 (40%)	n = 97
Demographic Characteristics	**Sex**				
	Male	28 (72%)	11 (58%)	17 (44%)	56 (58%)
	Female	11 (28%)	8 (42%)	22 (56%)	41 (42%)
	**Race**				
	Black or African American	22 (56%)	9 (47.5%)	28 (72%)	59 (61%)
	White	13 (33%)	9 (47.5%)	6 (15%)	28 (29%)
	Others	4 (11%)	1 (5%)	5 (13%)	10 (10%)
	**Ethnicity**				
	Non-Hispanic or Latino	24 (62%)	11 (58%)	31 (79%)	66 (68%)
	Hispanic or Latino	15 (38%)	8 (42%)	8 (21%)	31 (32%)
	**Age, years**				
	Mean (SD)	56 (9.07)	57 (8.27)	51 (10.51)	54 (9.75)
	Median (IQR)	58 (13.00)	60 (13.00)	53 (11.00)	56 (14.00)
Medical Characteristics	**Body Mass Index, kg/m^2^**				
	Mean (SD)	26.30 (4.94)	23.10 (3.07)	27.96 (6.00)	26.34 (5.37)
	Median (IQR)	25.63 (7.30)	22.86 (2.99)	28.53 (9.12)	25.06 (7.93)
	***CYP2B6* Allele**				
	Normal function	15 (39%)	7 (37%)	12 (31%)	34 (35%)
	Loss of function	22 (56%)	12 (63%)	26 (67%)	60 (62%)
	Gain of function	2 (5%)	0	1 (2%)	3 (3%)
	**Concomitant Medication**				
	No CYP2B6 inducer or inhibitor	37 (95%)	11 (58%)	39 (100%)	87 (90%)
	CYP2B6 Inducer	2 (5%)	8 (42%)	0	10 (10%)
	Fibrosis Stage*				
	F0-F2	22 (56%)	13 (68%)	33 (87%)	68 (71%)
	>F2	17 (44%)	6 (32%)	5 (13%)	28 (29%)
	Steatosis Level*				
	S0	25 (64%)	14 (74%)	19 (50%)	58 (61%)
	S1	4 (10%)	0	7 (18%)	11 (11%)
	S2-S3	10 (26%)	5 (26%)	12 (32%)	27 (28%)

*One participant had median vibration-controlled transient elastography values reported as 0, and is excluded from the calculation of descriptive statistics with respect to fibrosis stage and steatosis level. Steatosis levels S2 and S3 are combined since there are only 2 participants with S2.

Abbreviations: HCV, hepatitis C virus; CYP, cytochrome.

### Medical characteristics

For all participants, median BMI was 25.06 (IQR 7.93). Interestingly, uninfected individuals had the highest median BMI 28.53 (IQR 9.12), HCV mono-infected individuals had intermediate median BMI 25.63 (IQR 7.30), and HCV/HIV co-infected individuals had the lowest median BMI (22.86, IQR 2.99) of the three groups.

**Fig 1:**
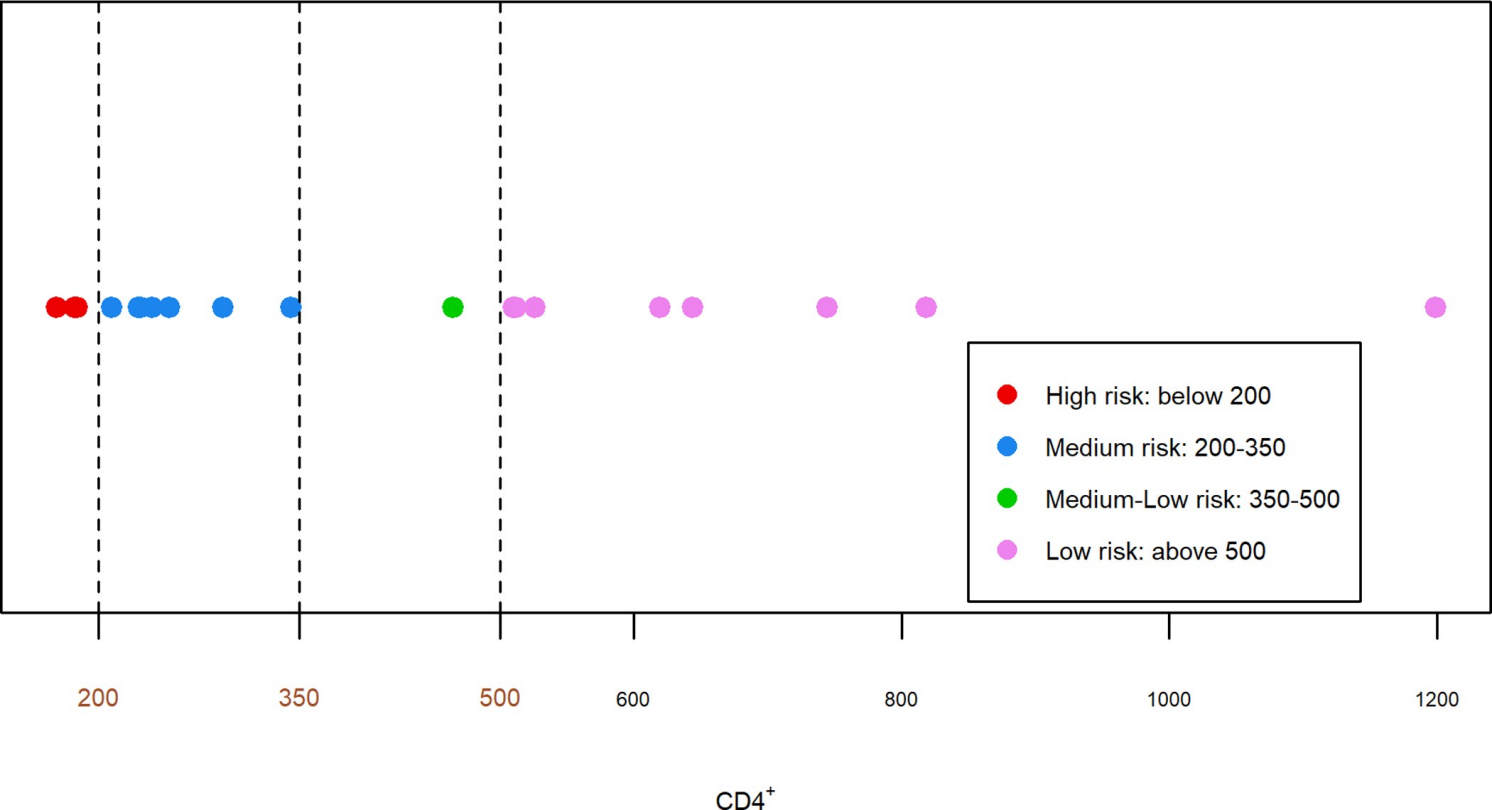
Severity of HIV disease based upon CD4^+^ cell count.

We categorized HCV/HIV co-infected participants’ CD4^+^ values according to treatment guidelines (Fig 1) [45]. Nine participants (47%) had undetectable HIV RNA, indicating their adherence with stable antiretroviral therapy.

Distribution of CD4^+^ cell counts (according to treatment guidelines) in hepatitis C virus (HCV)/HIV co-infected study participants. Individuals with CD4^+^ cell count <200 cells/mm^3^ are considered high risk for HIV disease progression. CD4^+^ cell counts between 200 and 350 cells/mm^3^ are at medium risk, CD4^+^ cell counts between 350 and 500 cells/mm^3^ are at medium-low risk, and those at low risk have CD4^+^ cell counts above 500 cells/mm^3^.

### Methadone metabolism measurements and correlations

The median methadone dose was 80 mg (IQR 60). Methadone dose did not differ by infection status (Table 3A). Methadone disposition and metabolism measurements are shown in Table 3A. When stratified by race, black individuals appeared to have slightly lower methadone doses although methadone metabolism did not appear to differ between blacks and whites or other races (Table 3B).

**Table 3:** **A.** Methadone disposition and methadone metabolism measurements. **B.** Methadone disposition and methadone metabolism measurements with respect to race. The table presents counts and associated percentages or mean, median and associated standard deviation or interquartile range as appropriate.

**A**					
	**Variable**	**Infection status**			
		**HCV-mono**	**HCV/HIV**	**Uninfected**	**Total**
		**n = 39** (40%)	**n = 19** (20%)	**n = 39** (40%)	**n = 97**
Methadone Disposition	**Methadone dose, mg**				
	Mean (SD)	92.56 (50.85)	85.53 (38.22)	95.64 (48.90)	92.42 (47.51)
	Median (IQR)	80.00 (72.50)	80.00 (50.00)	90.00 (72.50)	80.00 (60.00)
	**Dose normalized (R)-Methadone concentration, ng/ml/mg**				
	Mean (SD)	7.56 (5.66)	7.42 (4.67)	5.62 (2.29)	6.75 (4.44)
	Median (IQR)	6.06 (4.86)	6.02 (1.93)	5.61 (3.00)	5.96 (2.94)
	**Dose normalized (S)-Methadone concentration, ng/ml/mg**				
	Mean (SD)	6.91 (5.74)	6.86 (5.82)	5.96 (3.27)	6.51 (4.88)
	Median (IQR)	5.61 (4.98)	4.87 (2.74)	5.51 (4.30)	5.41 (4.41)
	**Dose normalized (R)-EDDP concentration, ng/ml/mg**				
	Mean (SD)	0.63 (0.59)	0.54 (0.40)	0.42 (0.18)	0.53 (0.43)
	Median (IQR)	0.45 (0.39)	0.42 (0.23)	0.40 (0.16)	0.42 (0.25)
	**Dose normalized (S)-EDDP concentration, ng/ml/mg**				
	Mean (SD)	0.81 (0.82)	0.72 (0.57)	0.58 (0.26)	0.70 (0.60)
	Median (IQR)	0.56 (0.49)	0.57 (0.34)	0.55 (0.23)	0.56 (0.32)
Methadone Metabolism	**(R)-EDDP/(R)-methadone concentration**				
	Mean (SD)	0.09 (0.05)	0.07 (0.03)	0.08 (0.03)	0.08 (0.04)
	Median (IQR)	0.08 (0.04)	0.07 (0.02)	0.08 (0.03)	0.08 (0.03)
	**(S)-EDDP/(S)-methadone concentration**				
	Mean (SD)	0.14 (0.10)	0.11 (0.04)	0.12 (0.05)	0.12 (0.07)
	Median (IQR)	0.12 (0.09)	0.11 (0.04)	0.11 (0.07)	0.11 (0.07)
**B**					
				**Black or African American**	**White or other races**
				**n = 59 (61%)**	**n = 38 (39%)**
Methadone Disposition		**Methadone dose, mg**			
		Mean (SD)		86.69 (42.14)	101.32 (54.22)
		Median (IQR)		80.00 (57.50)	87.50 (76.25)
		**Enantiomer methadone dose (Free Base), mg**			
		Mean (SD)		38.77 (18.85)	45.31 (24.25)
		Median (IQR)		35.78 (25.72)	39.13 (34.10)
Methadone Metabolism		**(R)-EDDP/(R)-methadone concentration**			
		Mean (SD)		0.08 (0.03)	0.09 (0.04)
		Median (IQR)		0.08 (0.04)	0.08 (0.03)
		**(S)-EDDP/(S)-methadone concentration**			
		Mean (SD)		0.12 (0.06)	0.14 (0.09)
		Median (IQR)		0.11 (0.06)	0.12 (0.08)

Abbreviations: HCV, hepatitis C virus; EDDP, 2-ethylidene-1,5-dimethyl-3,3-diphenylpyrrolidine; SD, standard deviation; IQR, interquartile range.

There were strong positive correlations between (R)-EDDP/methadone concentration and (S)-EDDP/methadone concentration ratios across the three infection statuses (Spearman’s correlation coefficient: HCV mono-infection = 0.91, HCV/HIV = 0.82, uninfected = 0.89). Similar patterns were observed between dose-normalized (R)- and (S)-methadone concentrations across infection status (Spearman’s correlation coefficient: HCV mono-infection = 0.94, HCV/HIV = 0.91, uninfected = 0.88). There were strong negative correlations between dose-normalized (R)- and (S)-methadone concentrations and (R)- and (S)-EDDP/methadone concentration ratios within uninfected participants (Spearman’s correlation coefficients -0.61 and -0.75, respectively). Hence, uninfected participants with lower methadone metabolism had higher methadone plasma concentrations.

### Methadone withdrawal and overdose symptomatology

We assessed symptoms of methadone withdrawal and overdose using the COWS and OOA, respectively. Of the 97 participants in this study, 96 responded to the COWS and all had scores below 5, indicating the absence of withdrawal symptoms. A total of 85 participants provided responses to the OOA. Of the patient reported symptoms, scores ranged from 0 to 16 out of a total symptom score of 32. In terms of signs, no participant had a decrease in respiratory rate compared to normal, 36 (42%) had pinpoint pupils, 2 (2.4%) were dozing, and 6 (7.1%) were drowsy.

### Fibrosis and steatosis

#### Fibrosis

The majority of participants had no, mild, or moderate fibrosis (71%, F0-F2; Table 2). Of those with fibrosis (>F2), 17 were HCV mono-infected, 6 were HCV/HIV co-infected, and 5 were uninfected.

#### Steatosis

A total of 58 (61%) participants had no steatosis (S0), 11 (11%) had mild steatosis (S1), and 27 (28%) had moderate to severe steatosis (S2-S3) (Table 2). Of the 27 participants with moderate to severe steatosis, 12 were uninfected, 10 were HCV mono-infected, and 5 were HCV/HIV co-infected.

#### Steatosis and BMI

**Fig 2:**
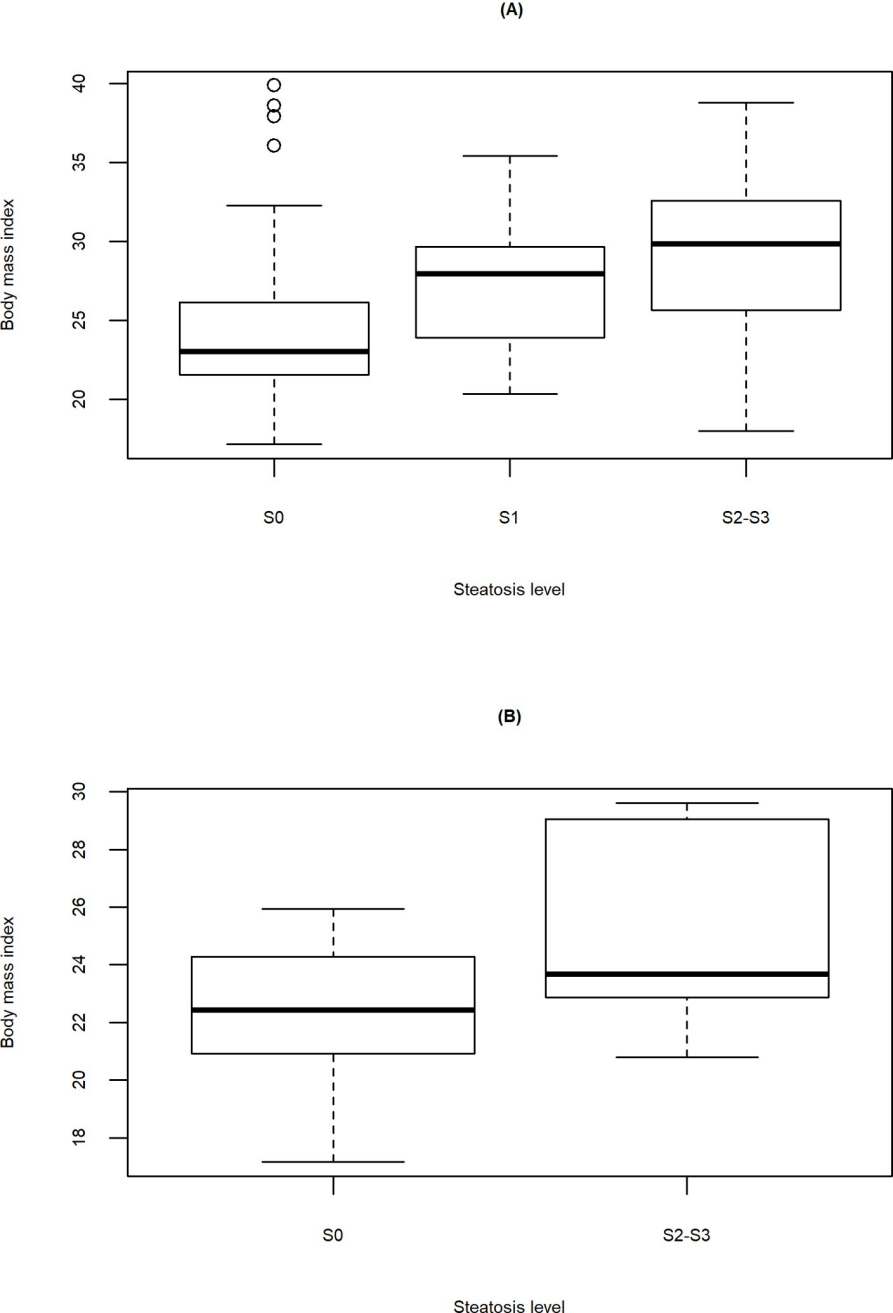
Body mass index by steatosis. Boxplots illustrate an increased trend of steatosis severity as body mass index increases for (A) all study participants and (B) HCV/HIV co-infected participants. S0 indicates healthy individuals, S1 indicates individuals with mild steatosis, and S2-S3 indicates individuals with medium or severe steatosis.

The distribution of BMI with respect to different levels of steatosis for all participants and HCV/HIV co-infected participants are illustrated in Fig 2A and 2B. As illustrated, the participants with higher BMI values had more advanced steatosis.

### CYP2B6 genotype

Recent data establish that CYP2B6 is the enzyme responsible for methadone metabolism to EDDP. *CYP2B6* alleles encode variant enzymes with LOF (e.g., CYP2B6*6, CYP2B6*16, CYP2B6*18) and gain-of-function (CYP2B6*4) compared to normal function (CYP2B6*1). Methadone metabolism by the variant gene product CYP2B6*6 is less than, while CYP2B6*4 is greater than, CYP2B6*1 [15, 46]. The study participants were represented by 9 different genotypes (Table 4).

**Table 4:** Distribution of single nucleotide polymorphisms and relationship to *CYP2B6* genotype among study participants.

Genotype	rs3745274 genotype (516G>T)	rs2279343 genotype (785A>G)	rs28399499 genotype (983T>C)	rs3211371 genotype (1459C>T)	Count (Frequency)
*1/*1	GG	AA	TT	CC	24(25%)
*1/*4	GG	GA	TT	CC	1(1%)
*1/*5	GG	AA	TT	TC	3(3%)
*1/*6	GT	GA	TT	CC	33(34%)
*1/*7	GT	GA	TT	TC	7(7%)
*1/*18	GG	AA	CT	CC	9(9%)
*4/*6	GT	GG	TT	CC	2(2%)
*6/*16	GT	GA	CT	CC	6(6%)
*6/*6	TT	GG	TT	CC	12(13%)

Subsequently, participants were categorized into 3 groups according to their *CYP2B6* genotype function on methadone metabolism: normal function (*1/*1, *1/*5 and *1/*7), LOF (*1/*18, *1/*6, *6/*6 and *6/*16) and gain of function (*1/*4 and *4/*6). The most prevalent CYP2B6 phenotype was LOF (62%), followed by normal function (35%) and lastly, gain of function (3%). The most prevalent *CYP2B6* genotype was *1/*6 (34%), followed by *1/*1 (25%) and *6/*6 (13%).

**Fig 3:**
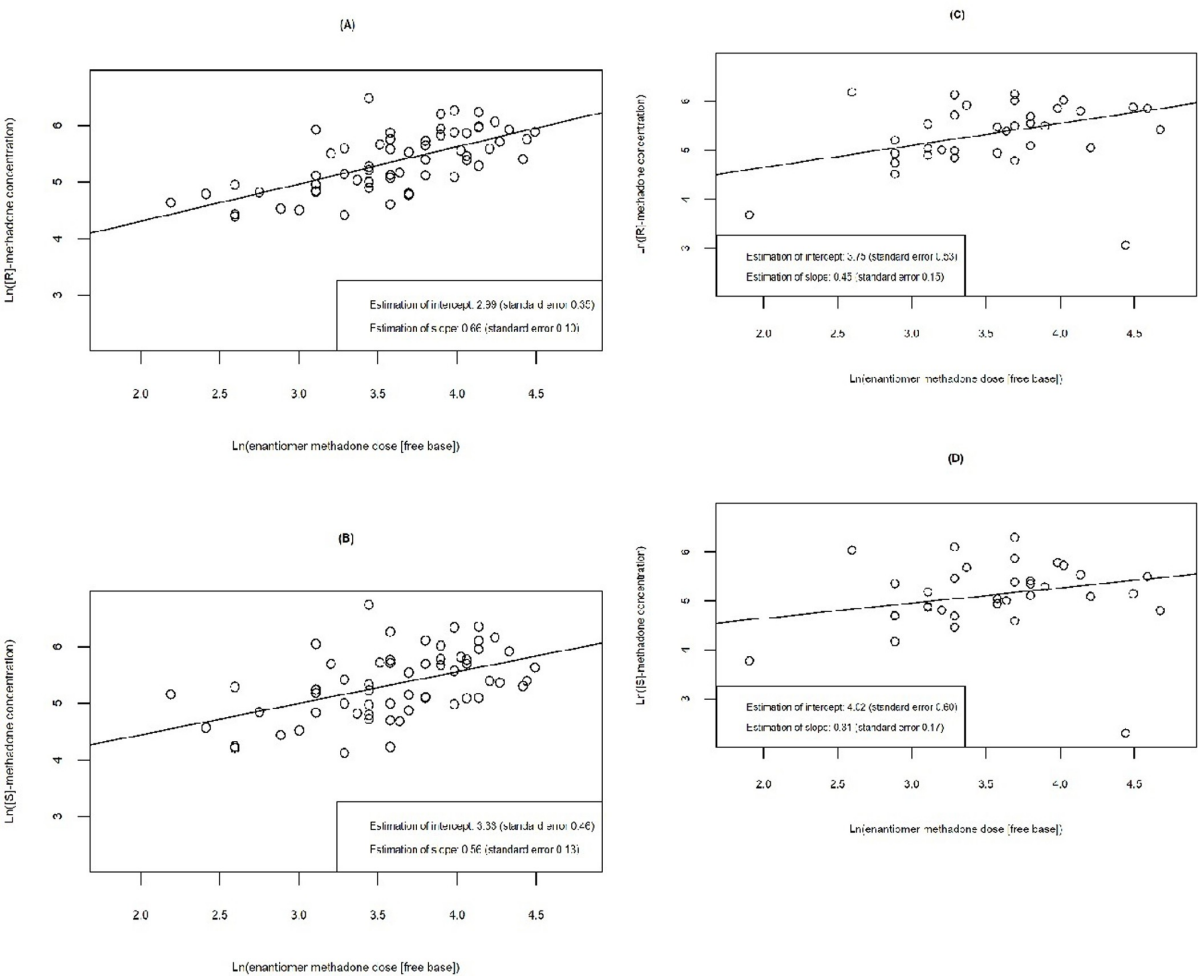
Linear relationship between ln(methadone concentration) and ln(methadone dose) by enantiomer and *CYP2B6* genotype function. In both LOF and normal function participants, (R)- and (S)-methadone concentration increases by 2.71 mg per increase in enantiomer methadone dose [i.e., one unit increase in ln(enantiomer methadone dose)]. In LOF participants, (A) average (R)-methadone concentration increases by 1.93 ng/ml [ln(R-methadone concentration) increases by 0.66, standard error 0.10], and (B) average (S)-methadone concentration increases by 1.75 ng/ml [ln(S-methadone concentration) increases by 0.56, standard error 0.13]. In normal function participants, (C) average (R)-methadone concentration increases by 1.57 ng/ml [ln(R-methadone concentration) increases by 0.45, standard error 0.15] and (D) average (S)-methadone concentration increases by 1.36 ng/ml [ln(S-methadone concentration) increases by 0.31, standard error 0.17].

We evaluated the relationship between methadone concentration and enantiomer methadone dose (free base) for each enantiomer and *CYP* genotype function categorized by normal function and LOF (Fig 3A–3D). We observed that LOF participants had greater increases in both (R)- and (S)- methadone concentration with increasing methadone dose compared with those with normal function genotype. For both genotypes, the increase in (R)-methadone concentration was higher than (S)-methadone concentration when methadone dose increased by the same unit.

**Fig 4:**
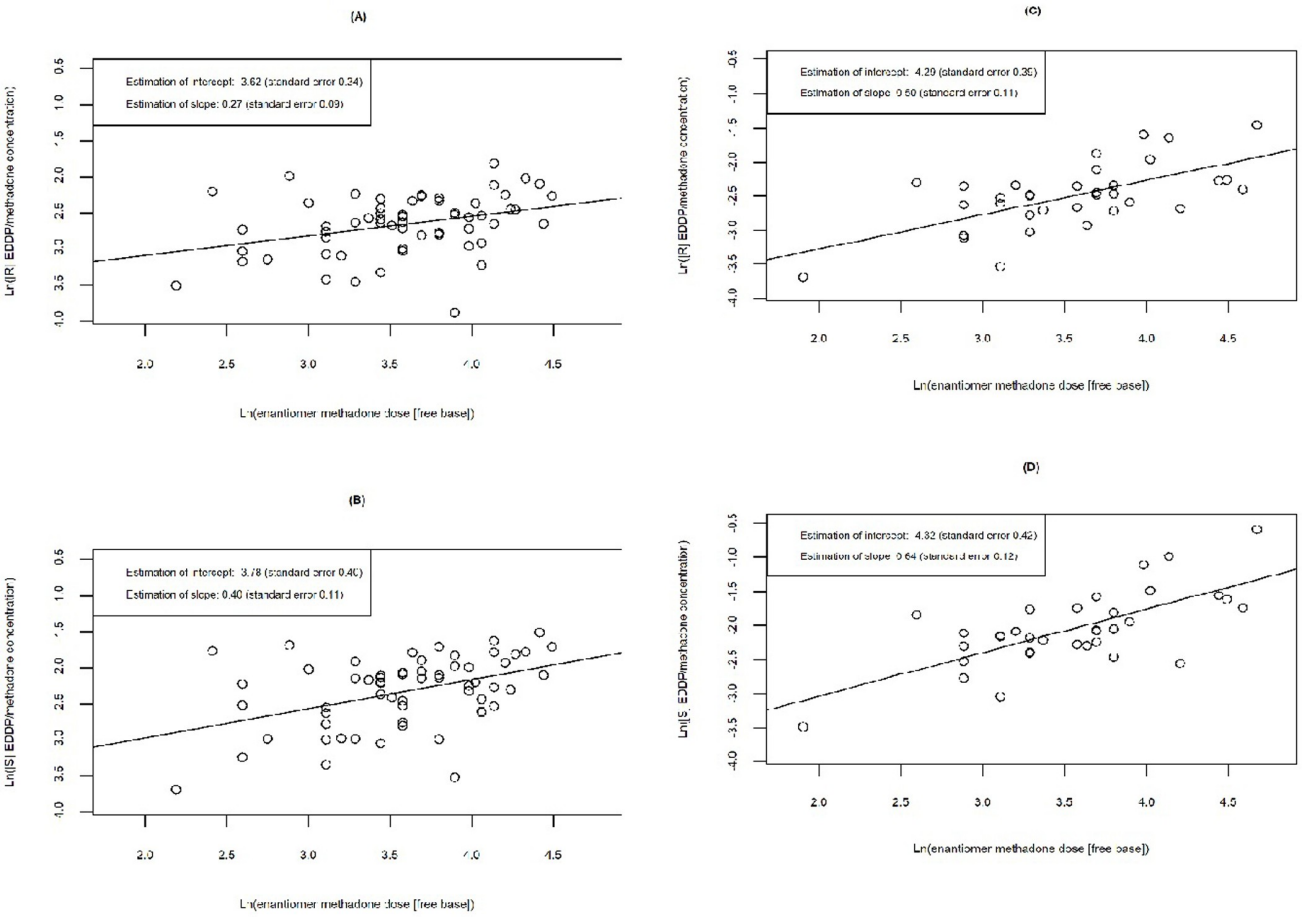
Linear relationship between ln(methadone metabolism) and ln(methadone dose) by enantiomer and *CYP2B6* genotype function. In both LOF and normal function participants, (R)- and (S)-EDDP/methadone concentration increases by 2.71 mg per increase in enantiomer methadone dose [i.e., one unit increase in ln(enantiomer methadone dose)]. In LOF participants, (A) average (R)-EDDP/methadone concentration increases by 1.31 [ln(R-EDDP/methadone concentration) increases by 0.27, standard error 0.09], and (B) average (S)-EDDP/methadone concentration increases by 1.49 [ln(S-EDDP/methadone concentration) increases by 0.40, standard error 0.11]. In normal function participants, (C) average (R)-EDDP/methadone concentration increases by 1.65 [ln(R-EDDP/methadone concentration) increases by 0.50, standard error 0.11], and (D) average (S)-EDDP/methadone concentration increases by 1.90 [ln(S-EDDP/methadone concentration) increases by 0.64, standard error 0.12].

We also evaluated the relationship between (R)- and (S)-methadone metabolism with increasing methadone dose for normal function and LOF participants (Fig 4A–4D). We observed greater increases in both (R)- and (S)-EDDP/methadone concentration in participants with normal function genotypes compared to LOF participants. These results suggest that participants with *CYP* 2B6 LOF genotypes have reduced methadone metabolism. In both normal and LOF participants, the increase in (S)-methadone metabolism was higher than (R)-methadone metabolism when the enantiomer methadone dose increased by the same unit.

We also stratified the *CYP2B6* genotypes by race, ethnicity, and sex (Table 5). The *CYP2B6* genotype *6/*16 was found in 6 participants (6%). Participants with this genotype were female (67%), African American (100%) and non-Hispanic (100%). The *CYP2B6* genotype *1/*18 was found in 9 participants (9%). The majority of participants with this genotype were male (89%), African American (78%) and non-Hispanic (67%). Furthermore, other genotypes (e.g., *1/*5, *1/*7) appeared to segregate by race and ethnicity. However, due to the small sample size, more information is needed to determine whether the frequency of these genotypes differ by race and/or ethnicity.

**Table 5:** Distribution of participants with respect to genotype, genotype by race, genotype by ethnicity and genotype by sex. Column percentages of genotypes are calculated with reference to the total number (n = 97) of participants. Row percentages are calculated with reference to the number of participants with each genotype.

Function	Genotype		Race			Ethnicity		Sex	
			Black or African American	White	Others	Non-Hispanic or Latino	Hispanic or Latino	Male	Female
Normal Function	*1/*1	24 (25%)	14 (58%)	9 (38%)	1 (4%)	16 (67%)	8 (33%)	13 (54%)	11(46%)
	*1/*5	3 (3%)	3 (100%)	0 (0%)	0 (0%)	3 (100%)	0 (0%)	2 (67%)	1(33%)
	*1/*7	7 (7%)	1 (14%)	5 (72%)	1 (14%)	3 (43%)	4 (57%)	1 (14%)	6(86%)
	Total	34	18	14	2	22	12	16	18
Gain of Function	*1/*4	1 (1%)	1 (100%)	0 (0%)	0 (0%)	1 (100%)	0 (0%)	0 (0%)	1(100%)
	*4/*6	2 (2%)	0 (0%)	2 (100%)	0 (0%)	1 (50%)	1 (50%)	1 (50%)	1 (50%)
	Total	3	1	2	0	2	1	1	2
Loss of Function	*1/*18	9 (9%)	7 (78%)	1 (11%)	1 (11%)	6 (67%)	3 (33%)	8 (89%)	1 (11%)
	*1/*6	33 (34%)	20 (61%)	7 (21%)	6 (18%)	23 (70%)	10 (30%)	21 (64%)	12(36%)
	*6/*16	6 (6%)	6 (100%)	0 (0%)	0 (0%)	6 (100%)	0 (0%)	2 (33%)	4 (67%)
	*6/*6	12 (13%)	7 (58%)	4 (34%)	1 (8%)	7 (58%)	5 (42%)	8 (67%)	4 (33%)
	Total	60	40	12	8	42	18	39	21

### Concomitant CYP2B6-inducer medications

To evaluate the effects of CYP2B6 inducer medications on methadone metabolism, participants were categorized into two groups: those taking or not taking CYP2B6 inducer medications. Ten participants were on a CYP2B6-inducing medication (10%): 1 participant was on carbamazepine, 6 were on efavirenz, and 3 were on nelfinavir. Among the HCV/HIV co-infected group, 8 participants were on a CYP2B6-inducing medication (42%).

**Fig 5:**
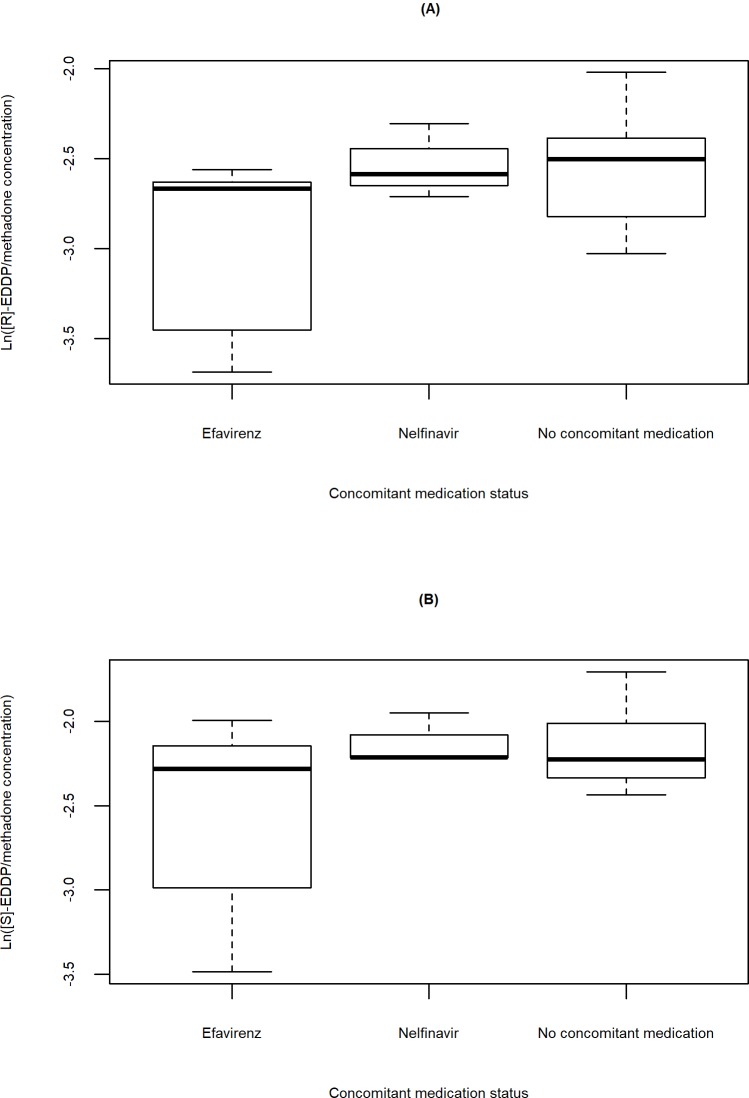
Methadone metabolism stratified by concomitant medication status. Boxplots of (A) ln (R)- and (B) ln (S)-EDDP/methadone concentration versus CYP2B6 inducer or no concomitant medication for HCV/HIV co-infected participants. Most of the participants who took CYP2B6 inducer medications were HCV/HIV co-infected. The lower ln([R]-EDDP/methadone concentration) and ln([S]-EDDP/methadone concentration) values are largely driven by efavirenz.

Ln([R]-EDDP/methadone concentration) and ln([S]-EDDP/methadone concentration) for HCV/HIV co-infected participants on a CYP2B6-inducing medication versus those not on a CYP2B6 inducer medication is illustrated in Fig 5A and 5B. Nelfinavir did not appear to have an effect on methadone metabolism. In contrast, methadone metabolism appeared to be inhibited in the five participants on efavirenz. Of note, the HCV mono-infected participant on carbamazepine had much higher values of ln([R]-EDDP/methadone concentration) and ln([S]-EDDP/methadone concentration) than the median values of participants who took other CYP2B6 inducer medications and participants with no concomitant CYP2B6 inducer medications. Since only one participant was prescribed carbamazepine, insufficient data exist to assess the full impact of the medication on methadone metabolism. Interestingly, the HCV/HIV co-infected participants on CYP2B6 inducer medications all had CD4^+^ cell counts of greater than 200 cells/mm^3^.

### Overall modeling results

Modeling results reveal that female sex has a significant effect (p = 0.016) on ln([R]-EDDP/methadone concentration), and on ln([S]-EDDP/methadone concentration) (p = 0.044), and consequently on (R)- and (S)-methadone metabolism. LOF alleles are significant (p = 0.012) on ln([S]-EDDP/methadone concentration), and therefore on (S)-methadone metabolism. BMI has a significant negative effect on ln([R]-EDDP/methadone concentration) (p = 0.034) and hence on (R)-methadone metabolism. Methadone metabolism appears to be lower in males, individuals with LOF alleles, and elevated BMI. Gain of function alleles and the interaction between LOF alleles and concomitant CYP2B6 inducer medications had no significant effects on methadone metabolism. The estimated fixed effects intercept for both ln([R]-EDDP/methadone concentration) and ln([S]-EDDP/methadone concentration) were significant (p<0.0001, Table 6). Table 6 presents the estimated coefficients of the fixed effects with their associated standard errors and the corresponding 95% confidence intervals.

**Table 6:** Estimates of coefficients for fixed effects and their associated 95% confidence intervals.

**Ln([R]-EDDP/methadone concentration):**					
**Effect**		**Estimate**	**Standard Error**	**Pr > |t|**	**95% Confidence Interval**
**Intercept**		-2.138	0.247	< .0001	(-2.626, -1.651)
**Sex**	**Female**	0.223	0.092	0.016	(0.042, 0.403)
	**Male**	0	.	.	.
**BMI**		-0.018	0.008	0.034	(-0.034, -0.001)
**Genotype**	**Gain of function**	-0.043	0.248	0.863	(-0.533, 0.447)
	**Loss of function**	-0.169	0.093	0.070	(-0.351, 0.014)
	**Normal function**	0	.	.	.
**Concomitant medication**	**Inducer Medication**	-0.175	0.209	0.403	(-0.588, 0.238)
	**No Medication**^a^	0	.	.	.
Genotype*Concomitant medication	Gain of function*No Medication	0	.	.	.
	Loss of function *Inducer Medication	0.175	0.276	0.527	(-0.370, 0.719)
	Loss of function *No Medication^a^	0	.	.	.
	Normal function * Inducer Medication	0	.	.	.
	Normal function * No Medication^a^	0	.	.	.
**Ln([S]-EDDP/methadone concentration):**					
**Effect**		**Estimate**	**Standard Error**	**Pr > |t|**	**95% Confidence Interval**
**Intercept**		-1.785	0.298	< .0001	(-2.373, -1.198)
**Sex**	**Female**	0.230	0.113	0.044	(0.006, 0.454)
	**Male**	0	.	.	.
**BMI**		-0.014	0.010	0.160	(-0.034, 0.006)
**Genotype**	**Gain of function**	-0.050	0.308	0.870	(-0.658, 0.557)
	**Loss of function**	-0.294	0.116	0.012	(-0.522, -0.065)
	**Normal function**	0	.	.	.
**Concomitant medication**	**Inducer Medication**	-0.144	0.256	0.575	(-0.648, 0.361)
	**No Medication**^a^	0	.	.	.
Genotype*Concomitant medication	Gain of function*No Medication	0	.	.	.
	Loss of function *Inducer Medication	0.263	0.345	0.447	(-0.417, 0.943)
	Loss of function *No Medication^a^	0	.	.	.
	Normal function * Inducer Medication	0	.	.	.
	Normal function * No Medication^a^	0	.	.	.

^a^NoCYP2B6 inducer or inhibitor medication.

Abbreviations: BMI, body mass index; EDDP, 2-ethylidene-1,5-dimethyl-3,3-diphenylpyrrolidine.

### Interpretation of significant parameters

#### Sex

For (R)-methadone, females have an estimated average 0.223 increase in ln([R]-EDDP/methadone concentration) compared with males, i.e. 1.250 increase in (R)-EDDP/methadone concentration. For (S)-methadone, females have an estimated average 0.230 increase in ln([S]-EDDP/methadone concentration) compared with males, i.e. 1.259 increase in (S)-EDDP/methadone concentration.

#### LOF alleles

Participants with LOF alleles have an estimated average 0.294 lower ln([S]-EDDP/methadone concentration), i.e. 1.342 lower S-EDDP/methadone concentration than the individuals with normal function alleles.

#### BMI

For (R)-methadone, when BMI of the participant increases 1 kg/m^2^, the estimated average ln([R]-EDDP/methadone concentration) will decrease by 0.018, i.e. the (R)-EDDP/methadone concentration decreases by 1.018.

#### Random effects

The estimated variance of the site random effect is 0.04986 and the one associated with the infection status random effect (nested within sites) is 0.005519.

#### CYP2B6 inducer medications

We noticed a negative estimated coefficient of inducer medication group, -0.175 with standard error of 0.209 for ln([R]-EDDP/methadone concentration) and, similarly, -0.144 with standard error of 0.256 for ln([S]-EDDP/methadone concentration). This may be due to the presence of subgroups and population heterogeneity (there is a strong interaction between infection status and concomitant medication variables with a p-value of 3.13x10^-6^ and an I^2^ statistic of 95.4%).

#### Intercept

For both ln([R]-EDDP/methadone concentration) and ln([S]-EDDP/methadone concentration), the estimated intercepts are significant. The intercept indicates the estimated average value of ln([R]-EDDP/methadone concentration) or ln([S]-EDDP/methadone concentration) when an individual has baseline characteristics (male, normal function CYP2B6, BMI equal to 0 and takes no concomitant CYP2B6 inducer medications).

## Discussion

Methadone’s narrow therapeutic index and wide inter-individual PK variability create dosing challenges; these challenges frequently lead to sub-therapeutic plasma concentrations or to the consequences of overdose. We evaluated the factors contributory to methadone metabolism. We found that (R)- and (S)- methadone metabolism was significantly affected by sex; (S)-methadone metabolism was significantly affected by *CYP2B6* LOF alleles. BMI was also significant for (R)-methadone metabolism. Additionally, CYP2B6 inducer medications did not have a significant effect on methadone metabolism.

The role of the hepatic enzyme primarily responsible for methadone metabolism has been controversial and an active area of investigation since methadone was approved for treatment of opioid addiction by the United States Food and Drug Administration in 1972. Early in vitro studies concluded that methadone was mainly catalyzed by CYP3A4, although clinical data were lacking [47]. Indeed, CYP3A4 genetic polymorphisms were not predictive of methadone dose [48]. The US Food and Drug Administration-approved label indicates that CYP P450 enzymes, primarily CYP3A4, CYP2B6, CYP2C19, CYP2C9, and CYP2D6, are responsible for methadone metabolism [49]. According to recent in vitro and *in vivo* studies, CYP2B6 inhibition decreased methadone N-demethylation and increased plasma methadone concentrations. In contrast, CYP3A4 inhibition consistently failed to decrease methadone metabolism and clearance. Thus, CYP induction and inhibition studies support a more prominent role for CYP2B6 rather than CYP3A4 in methadone N-demethylation, clearance and plasma concentrations [12, 30, 50–52]. *CYP2B6* polymorphisms may be critical for methadone’s inter-individual PK variability. However, evaluation of the frequency and effects of *CYP2B6* alleles in individuals chronically maintained on methadone is limited. We divided the *CYP2B6* alleles in to three groups based upon their relationship to methadone metabolism: normal function, LOF or gain of function. Interestingly, the most common alleles in this largely racial and ethnic minority population were associated with LOF. With regard to methadone enantiomers, CYP2B6 metabolizes (S)-methadone to a greater extent than the (R)-methadone enantiomer [10, 50]. We observed greater increases in (S)- than (R)- methadone metabolism per unit increase in methadone dose consistent with these prior observations. Furthermore, the propensity for (S)-methadone metabolism by CYP2B6 may explain our observation of a significant effect of LOF alleles on (S)-methadone metabolism while their effect on (R)-methadone metabolism was not significant. We did not observe an association between gain of function *CYP2B6* alleles and altered methadone metabolism, although small numbers of study participants (n = 3) with these alleles may have impacted our results. Larger studies are needed to assess the effect of these alleles on methadone metabolism.

We were able to stratify *CYP2B6* alleles by race and ethnicity. Consistent with prior observations, we noted that *6/*16 and *1/*18 genotypes were almost exclusively found among our study participants of African origin [53]. While investigation of methadone metabolism by *6/*16 has not been performed, the allele has been noted to be an important factor in efavirenz metabolism by CYP2B6 in African-origin individuals [54]. Furthermore, the *6 allele, which results in decreased methadone metabolism, is common in Africans, Hispanics, and Asians [11, 24, 55, 56]. We observed that *1/*6 was the most common genotype in our participants, occurring largely in African Americans. Other genotypes also appeared to segregate according to Caucasian or African origin, although small numbers of study participants prohibited further evaluation of these observations. Indeed, the effects of genetic polymorphisms on methadone metabolism have been characterized in Han Chinese and in individuals from Germany and Switzerland [57–59]. According to pharmacokinetic studies based on race, methadone N-demethylation and clearance were significantly lower in African Americans than Caucasians due to the proportionally greater number of CYP2B6*6 carriers and the absence of CYP2B6*4 carriers in African Americans [11, 24]. However, additional studies of *CYP2B6* polymorphism in African Americans have not been conducted. These observations highlight the importance of pursing genetic investigation in African-origin populations. For example, pharmacogenomics is extremely important in precision prescribing of medications with the goal of preventing adverse events and improving therapeutic effectiveness through dosage optimization. This has been attempted with warfarin, a commonly used anticoagulant that is inherently difficult to dose correctly. However, due to a lack of clinical studies in persons of African ancestry, pharmacogenetics is not currently recommended when prescribing warfarin for this population [60]. Inclusion of individuals of diverse ancestry can also improve discovery for complex traits that may only apply to minority populations [61].

Sex had a significant effect on both (R)- and (S)-methadone metabolism. Conflicting data exist on the role of sex in methadone metabolism, largely due to small sample sizes and a lack of racial/ethnic diversity in populations studied. For example, de Vos *et al*. [62] demonstrated a trend toward slower methadone elimination in women than in men (n = 20). Foster *et al*. [63] observed no differences between males and females in unbound fractions of (R)- and (S)-methadone (n = 18), and Preston *et al*. [64] found that the EDDP/methadone ratio did not differ by sex, but EDDP concentration was higher in women than in men (n = 19). We also evaluated the effect of CYP2B6 inducer medications, namely nelfinavir and efavirenz, on methadone metabolism. Efavirenz had substantial heterogeneity in its effect, although the small number of participants precludes us from definitive conclusions. Our results highlight the need for a large-scale study on sex differences in methadone metabolism, especially in racial and ethnic minority populations.

We also observed a significant result between methadone metabolism and BMI. Although our data are cross-sectional, study participants were on long-term, steady-state methadone. Additionally, methadone has been shown to result in medication-induced weight gain [65]. We did not observe a relationship between either fibrosis or steatosis and methadone concentration or metabolism. We assessed fibrosis utilizing VCTE to assess liver stiffness, a surrogate for liver fibrosis, and the continuous attenuation parameter, a measure of hepatic steatosis. Cirrhosis reduces hepatic medication metabolism, and we sought to utilize VCTE as a method to assess the effect of hepatic insufficiency on drug metabolism [66]. As expected, a higher percentage of those with HCV infection had at least moderate fibrosis (>F2) compared to the uninfected participants. Among all participants, the percentage of those with the most severe steatosis (S2-S3) was roughly equivalent between the HCV mono-, HCV/HIV co-, and uninfected participants. Median BMI was highest in the uninfected participants and lowest in HCV/HIV co-infection. While HIV and antiretroviral agents have been implicated in steatosis development [21], other etiologies, such as diabetes and the metabolic syndrome, may be the leading causes of hepatic steatosis in uninfected individuals. We also found a positive association between BMI and steatosis. In summary, advanced fibrosis occurred most frequently among participants with viral infection (HIV/HCV co- and HCV mono-infection), while BMI levels were lowest in those with HIV/HCV co-infection.

The relationship between HCV infection, liver fibrosis, and methadone metabolism has been evaluated in Eastern European and Taiwanese populations. Kljucevic *et al*. [67] observed in a Croatian population with HCV infection that the urine EDDP/methadone ratio and plasma EDDP concentration significantly decreased in a linear fashion with increasing fibrosis stage. Therefore, the authors concluded that liver damage decreases methadone metabolism. However, this investigation has several weaknesses including lack of clarity as to how HCV status was defined and determined, measurements limited to peak methadone concentration, a homogeneous participant population consisting of males of limited racial and ethnic diversity, and absence of cirrhotic participants [67]. In a Taiwanese population, Wu *et al*. [68] found that total plasma methadone and (R)-methadone concentrations were significantly higher in HCV seropositive compared with HCV seronegative participants. This study is limited since HCV status was only determined based upon antibody testing, which does not differentiate between active and resolved infections. Despite these limitations, both studies are in agreement that HCV influences plasma methadone concentration in OUD patients on methadone [67, 68]. In contrast to the above-mentioned studies, we did not observe a significant effect of fibrosis stage on methadone metabolism. However, we specifically assessed the presence of HCV RNA as a measure of active infection, and excluded those with inactive HCV infection.

While the importance of *CYP2B6* alleles responsible for methadone metabolism, especially in a minority population, can be considered a novel finding, the principal study limitation is the relatively small numbers of participants. The cross-sectional study design also limited our ability to investigate formal PK relationships, and we were limited to a single trough methadone measurement. Another limitation, albeit unexpected, is the presence and severity of steatosis and fibrosis in the uninfected participants as well as the lack of data assessing alcohol use and the presence of alcohol use disorder. However, this proof-of-concept study is innovative and timely as we were able to conduct translational research in an OTP, where OUD patients on methadone routinely congregate. Conducting research in venues where OUD patients feel more comfortable, such as in OTPs where they have care teams responsible for their treatment (e.g., social workers, counselors, nurses, health care providers) may facilitate research participation. We were able to leverage the comfortable and familiar environment within the OTP to engage OUD patients on methadone in a translational research study. An additional research consideration is that an OTP’s primary mission is clinical care. Indeed, the infrastructural and space requirements for research conduct, such as a separate room to accommodate VCTE examinations or laboratory space to accommodate specimen processing, can be problematic. Obtaining methadone concentration measurements from a largely minority population of OUD participants on methadone would have been infeasible without the ability to engage them in the clinical setting.

In summary, we identified the importance of LOF alleles, sex and BMI as determinants of methadone metabolism. Future studies should seek to understand the role of genotyping in accurate methadone dosing and evaluate methadone-antiretroviral agent interactions [8, 9]. In the age of precision medicine, genetic analysis is essential to deliver individualized treatments. Furthermore, it is crucial to include African-origin individuals in genetic studies as some *CYP2B6* alleles are race specific [53]. Our results also suggest that sex and *CYP2B6* genotype should be incorporated into multivariate models, along with other predictors (e.g., BMI), to create methadone dosing algorithms. The development of methadone-dosing algorithms could facilitate its delivery and improve patient satisfaction with methadone prescription as well as prevent overdose and death.

## Supporting information

S1 File Description of statistical modeling. This file contains an in depth description of the statistical modeling methods used in the manuscript.
(DOCX)

S2 File Supporting data. This file contains the data supporting the work described in the manuscript.
(XLSX)

S3 File Data example for SAS code. This file contains an example of the data used in the SAS code.
(CSV)

S4 File SAS code. This file contains the statistical code used in the manuscript (SAS).
(SAS)
