# Supplementary material for: Toward precision prescribing for methadone: Determinants of methadone deposition
Source: PLoS One. 2020 Apr 17;15(4):e0231467. doi: 10.1371/journal.pone.0231467 (PMC7164646; doi:10.1371/journal.pone.0231467)
Supplement: S1 File — This file contains an in depth description of the statistical modeling methods used in the manuscript. (DOCX) [file pone.0231467.s001.docx]

**Online Appendix**

Mixed Effects Model: We present details on the mixed effects model we use to model our data using SAS 9.4.

For $i^{th}$ participant with a given infection status $j$, from site $c$ , $X_{Sex, ijc}$ indicates the participant’s sex, where male is indicated by $X_{Sex, ijc}$=0 and female is indicated by $X_{Sex, ijc}$=1; $X_{BMI, ijc}$ presents the participant’s BMI value. The variable genotype has 3 categories; hence, it is represented by 2 variables, $X_{G1, ijc}$ and $X_{G2, ijc}$. Specifically, the “normal function” category is a reference category and for participants with loss of function (LOF) genotypes $X_{G1, ijc}$=1, $X_{G2, ijc}$=0. For participants with gain of function genotypes $X_{G1, ijc}=0, X_{G2, ijc}$=1. Thus, the variables $X_{G1, ijc}$ and $X_{G2, ijc}$ represent either a LOF or gain of function genotype. $X_{CM, ijc}$indicates the participant’s concomitant CYP2B6 inducer medication status; the participants who take no concomitant CYP2B6 inducer medications have value 0 while participants who take CYP2B6 inducer medications have $X_{CM, ijc}=1$, i.e. a participant who takes no concomitant CYP2B6 inducer medications belongs to the reference category. $X_{G1CM, ijc}$ and $X_{G2CM, ijc}$ are the interactions of genotype and concomitant CYP2B6 inducer medication status, where $X_{G1CM, ijc}=X_{G1, ijc}\times X_{CM, ijc}, X_{G2CM, ijc}=X_{G2, ijc}\times X_{CM, ijc}$.

Therefore, for $i^{th}$ participant with a given infection status $j$, from site $c$, the multivariate mixed effects model is given as

$$Y_{̰ijc}={X̰}_{ijc}B+(\gamma_{c}+\lambda_{j\left( c \right)})1+\varepsilon_{̰i(jc)} ,$$

$$i=1,2,\ldots, n_{jc};j=1,2,3;c=1,2,\ldots, 6 ; \sum_{c=1}^{6} \sum_{j=1}^{3} n_{jc}=97,$$

where $Y_{̰ijc}$ is a $1\times2$ vector with elements $Y_{R, ijc}$ and $Y_{S, ijc}$ indicating the logarithm (with natural basis) of the R-EDDP and S-EDDP over the methadone concentration. The subscript $ijc$ indicates the $i^{th}$ participant with a given infection status $j$, from site $c$, and the summation of the number of participants from each combination of site and infection status equals to the total sample size 97. Hence, the vectors $Y_{̰ijc}$ denote the outcome variable for each individual. Additionally, ${X̰}_{ijc}$ is a $1\times8$ vector incorporating the covariates of interest for each individual, given as

${X̰}_{ijc}=\left( 1, X_{Sex, ijc}, {X_{BMI, ijc}, X}_{G1, ijc},X_{G2, ijc}, X_{CM, ijc}, X_{G1CM, ijc}, X_{G2CM, ijc} \right)$,

and $B$ is a matrix with dimensions $8\times2$, with columns $B_{R}$ and $B_{S}$ defined as

$${B̰}_{R}=\left( \beta_{0, R},\beta_{Sex, R}, \beta_{BMI, R}, \beta_{G1, R}, \beta_{G2, R}, \beta_{CM, R}, \beta_{G1CM, R}, \beta_{G2CM, R} \right)^{T},$$

$${B̰}_{S}=\left( \beta_{0, S},\beta_{Sex, S}, \beta_{BMI, S},\beta_{G1, S}, \beta_{G2, S}, \beta_{CM, S}, \beta_{G1CM, S}, \beta_{G2CM, S} \right)^{T},$$

are the parameter vectors associated with the covariates of interest for R- and S-methadone metabolism respectively. The subscript $i(jc)$ of the error term can be thought as the $i^{th}$ participant nested within the combination of site $c$ and $j^{th}$ level of infection status. The notation $\varepsilon_{̰i(jc)}$ indicates a $1\times2$ error vector with elements $\varepsilon_{R,i(jc)}$ and $\varepsilon_{S,i(jc)}$ and covariance matrix $V=\left( \begin{matrix} \sigma_{1}^{2} & \sigma_{12} \\ \sigma_{12} & \sigma_{2}^{2} \end{matrix} \right)$, $Var\left( \varepsilon_{R,i(jc)} \right)=\sigma_{1}^{2}$, $Var\left( \varepsilon_{S,i(jc)} \right)=\sigma_{2}^{2}$, and $\sigma_{12}$ denotes the covariance between $\varepsilon_{R,i(jc)}$ and $\varepsilon_{S,i(jc)}$. Finally, $1={(1,1)}^{T}$, $\gamma_{c}\sim N(0, \sigma_{3}^{2})$ and $\lambda_{j(c)}\sim N\left( 0, \sigma_{4}^{2} \right).$ That is,$\gamma_{c}$ denotes the random intercept of the site $c$, which the individual originates and $\lambda_{j(c)}$ denotes the random intercept of the $j^{th}$ level of infection status nested within the site $c$.

With the multivariate linear mixed effects model , we assume that 1) the explanatory variables sex, BMI, genotype, concomitant CYP2B6 inducer medication usage and the interaction term are related linearly to the response variables ln([R]-EDDP/methadone concentration) and ln([S]-EDDP/methadone concentration); 2) the residuals for each individual are independent, normally distributed, and have constant variance $\sigma_{1}^{2}$ in terms of ln([R]-EDDP/methadone concentration), and the residuals for each individual are independent, normally distributed, and have constant variance $\sigma_{2}^{2}$ in terms of ln([S]-EDDP/methadone concentration); 3) for each individual participant, the residuals of fitting ln([R]-EDDP/methadone concentration) and ln([S]-EDDP/methadone concentration) are correlated with the covariance $\sigma_{12}$, which captures the association between ln([R]-EDDP/methadone concentration) and ln([S]-EDDP/methadone concentration) within each participant; 4) the random intercepts of sites are independent, normally distributed, and have constant variance $\sigma_{3}^{2}$ for each participant for both ln([R]-EDDP/methadone concentration) and ln([S]-EDDP/methadone concentration); 5) the random intercepts of infection status nested in sites are independent, normally distributed, and have constant variance $\sigma_{4}^{2}$ for each participant for both ln([R]-EDDP/methadone concentration) and ln([S]-EDDP/methadone concentration). The significance level for all analyses was set at p <0.05.
